# Size distributions of droplets produced by ultrasonic nebulizers

**DOI:** 10.1038/s41598-019-42599-8

**Published:** 2019-04-16

**Authors:** Stefan Kooij, Alina Astefanei, Garry L. Corthals, Daniel Bonn

**Affiliations:** 1 Van der Waals-Zeeman Institute, University of Amsterdam, Science Park 904, Amsterdam, 1098 XH The Netherlands; 2 Van t Hoff Institute for Molecular Sciences, University of Amsterdam, Science Park 904, Amsterdam, 1098 XH The Netherlands

## Abstract

In many applications where small, similar-sized droplets are needed, ultrasonic nebulizers are employed. Little is known about the mechanism of nebulization, for example about what determines the median droplet size. Even less understood, is the droplet size distribution, which is often simply fitted with a log-normal distribution or assumed to be very narrow. We perform the first systematic study of droplet size distributions for different nebulizer technologies, showing that these distributions can be very well fitted with distributions found for sprays, where the size distribution is completely determined by the corrugation of ligaments and the distribution of ligament sizes. In our case, breakup is believed to be due to pinch-off of Faraday instabilities. The droplet size distribution is then set by the distribution of wavelengths of the standing capillary waves and the roughness of the pinch-off ligaments. We show that different nebulizer technologies produce different size distributions, which we relate to (variation in) wavelengths of the waves that contribute to the droplet formation. We further show that the median droplet size scales with the capillary wavelength, with a proportionality constant that depends only slightly on the type of nebulizer, despite order-of-magnitude differences in other parameters.

## Introduction

Ultrasonic nebulizers or atomizers are very important due to their many applications such as drug delivery, mass spectrometry, humidity control, spray pyrolysis, coating, etc. In almost all cases, the distribution of droplet sizes is an important parameter. For example, in many drug delivery systems, droplets need to be sufficiently small to reach the lower parts of the pulmonary tract, as larger droplets are deposited predominantly at the start of the airways^1^. Despite extensive research^2–12^, many aspects of the nebulization process are still poorly understood, mainly because of the very complicated dynamics and the small length and time scales on which it takes place. This work is the first study that focusses primarily on the droplet size distribution for ultrasonic nebulization. We show that, where direct measurements are mostly inaccessible, the shape of the size distribution can be used as an indirect measure for the breakup mechanism. Depending on the type of nebulizer, the mechanism of nebulization can be very different. This is reflected in the droplet sizes, that unlike what is commonly observed, can be both narrowly or broadly distributed.

**Figure 1:**
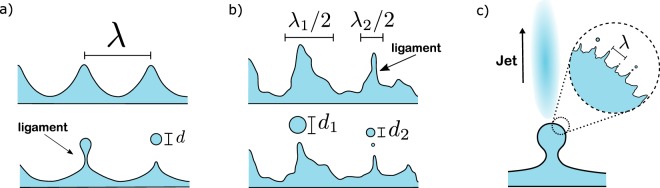
(**a**) Ideal case of standing capillary waves of wavelength *λ*. If the amplitude exceeds a certain value, equal sized ligaments are produced, leading to the break-off of monodisperse droplets of size *d* ~ *λ*. (**b**) Capillary waves in a system with maximum interference, leading to a distribution of wavelengths and amplitudes. This results in a broader distribution of droplet sizes set by the average ligament roughness and the distribution of ligament sizes. (**c**) Faraday waves are superposed on larger waves of the order of the wavelength of the chip material. The motion of these larger waves initiates breakup of the smaller superposed capillary waves. High-speed camera images show that jets of small droplets are formed at the crests of these larger waves at maximum acceleration.

The process of ultrasonic nebulization is mostly explained by the capillary wave mechanism. The formation of capillary waves on the surface of a fluid supported by a vibrating solid was first described by Faraday in 1831, and are therefore also referred to as Faraday waves^13^. Later, Kelvin derived the formula for the capillary wavelength *λ*^14^, relating it to the surface tension *σ*, density of the fluid $\rho$, fluid depth *h* and frequency *f* of the standing waves as follows:

1 $$\lambda {[\tanh (\frac{2\pi h}{\lambda })]}^{-1/3}={(\frac{2\pi \sigma }{\rho {f}^{2}})}^{1/3},$$

where for a sufficiently deep liquid layer, i.e. *h*/$\lambda \gg 1$, one can take $\tanh (2\pi h/\lambda )=1$. As with many parametric oscillators, the frequency of the nonlinear standing waves is half the excitation frequency *F*, i.e. $f=F$/2. For ultrasonic nebulizers, according to the capillary wave mechanism, the amplitude of the oscillation is large enough to cause droplet pinch-offs, thereby nebulizing the fluid (Fig. 1a). Since the size of the pinch-off droplets is proportional to the capillary wavelength, the median droplet size (designated as *D*_50_) is given by

2 $${D}_{50}=\kappa \lambda =\kappa \,{(\frac{8\pi \sigma }{\rho {F}^{2}})}^{1/3},$$

where *F* is the frequency of the nebulizer and $\kappa$ a proportionality constant. Lang was the first to experimentally determine this constant and found $\kappa =0.34$, where *D*_50_ is the median droplet size for the *number* (not *volume*) distribution^2^. For all that follows, we will use *D*_50_ only to indicate the median particle size by *volume*. Although Lang’s prediction works well in many cases, there are often slight and sometimes even significant differences in the proportionality constant $\kappa$^12,15^. This and the fact that many nebulizers are distinctly different has led to the formulation of many different scaling laws and different nebulization mechanisms, one example being cavitation^4,5,10^.

In studies of ultrasonic nebulizers, the focus is mostly on the median droplet size. The spread around this median is however just as important, especially for their implementation in different applications. For sprays and jets, the shape of the droplet size distribution is well understood^16–21^. There, it is set by the ligament corrugation and the distribution of ligament sizes. Assuming that capillary waves are the droplet formation mechanism, the droplet sizes are determined by the initial size of the waves and the roughness of the pinch-off ligaments (Fig. 1), and would therefore be comparable with the breakup of sprays. In this case, waves can be more or less spread in size, giving more or less dispersion in droplet sizes (Fig. 1b). Similarly, ligaments can be very corrugated, leading to a broad distribution, or very smooth giving a narrow size distribution.

In this work we investigate three types of ultrasonic nebulizers, working at different frequencies. We find that for these devices the capillary wave hypothesis works well, with proportionality constants depending on the type of nebulizer. We further show that droplet size distributions also depend on the type of nebulizer. Distributions can be surprisingly broad, presumed to be due to large variability in wavelengths and rough pinch-off ligaments, but also very narrow as predicted by the classical picture of capillary wave breakup, where waves are similar sized with smooth pinch-offs.

## Nebulizers

### SAWN

**Figure 2:**
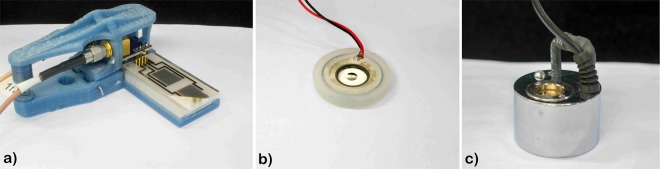
Nebulizer types investigated. (**a**) SAWN chip with holder. (**b**) Nebulizer chip. (**c**) Mist maker. The gold-colored part is the vibrating membrane. The transducer is placed roughly 4 cm under water.

**Figure 3:**
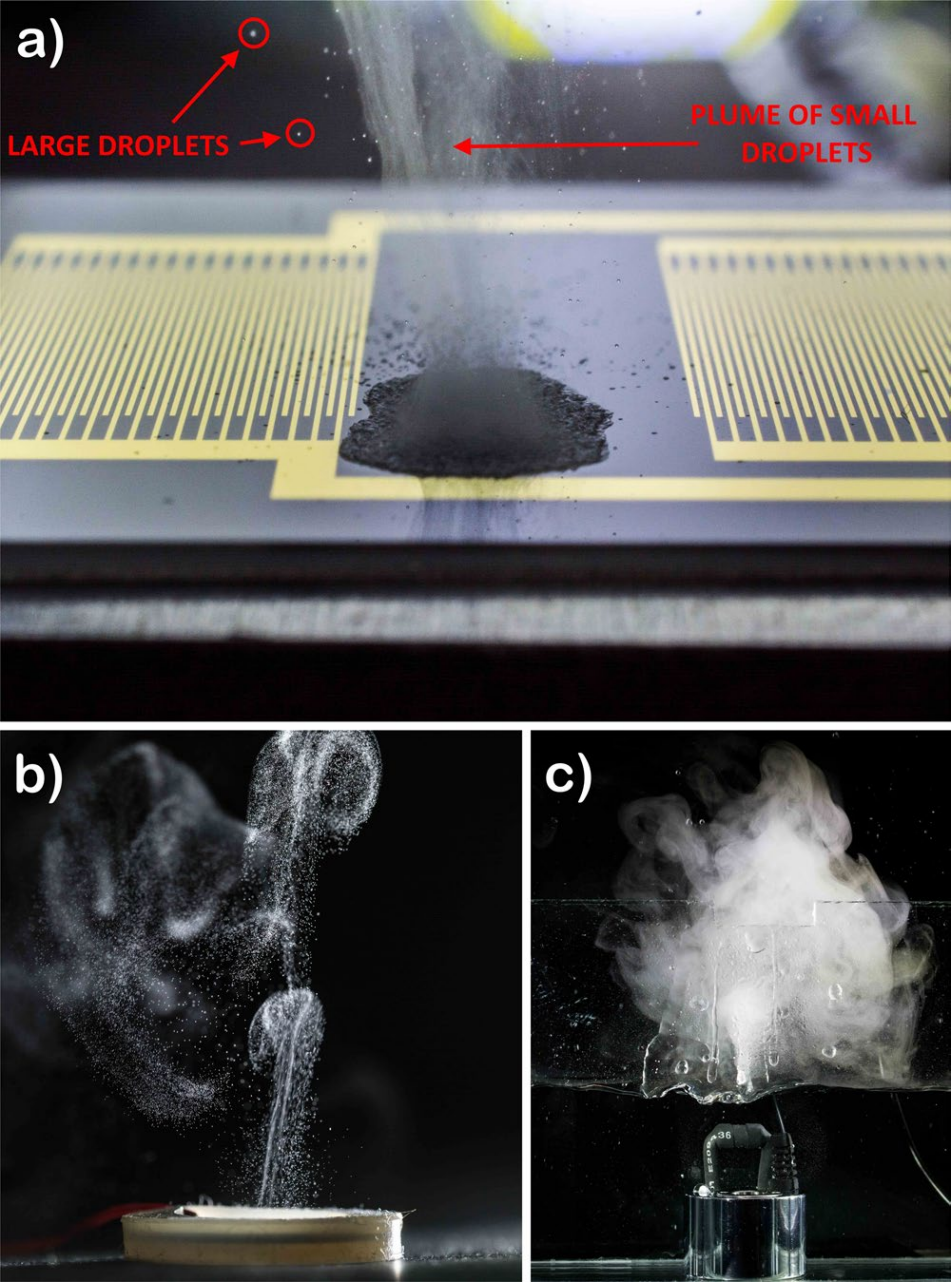
(**a**) Picture of the nebulization of a droplet on the SAWN chip taken with a 5 *μ*s flash light. Two droplet size regimes can be observed as indicated by the red arrows. (**b**) The nebulization process for the nebulizer chip. (**c**) The mist maker producing large amounts of small droplets. As can be seen, the mist maker is the only submerged nebulizer, where for the other nebulizers small amounts of fluids are placed on top of the transducer.

The surface acoustic wave nebulizer (SAWN) consists of two interdigital transducers (IDT) with lithium niobate as the piezoelectric substrate. Between the two IDTs there is a space (delay zone) where a droplet can be placed for nebulization (Fig. 2b with holder and Fig. 3a). For this particular chip, the resonance frequency is 9.6 MHz. The distance between the metallic electrodes of the IDT sets the wavelength of the acoustic waves at $\lambda =0.36\,{\rm{mm}}$. The surface acoustic waves can either be created by one of the IDTs (single mode) or both (dual mode). In this work, one IDT is used to create the waves and the other IDT as a transceiver. Experiments do not show any difference between the single and dual mode other than quicker nebulization for the latter.

The SAWN has many applications of which one of the most prominent ones is in mass spectrometry, where it has several advantages over more conventional nebulization methods^22,23^. The SAWN creates plumes of droplets with ionized molecules, which after the evaporation of the solvent, can be directly used by the mass spectrometer. The precise mechanism of the ionization is unknown, but is probably due to the high voltages present on the chip. It can also be a result of cavitation, which is known to produce sufficiently high temperatures and pressure^24^, but is considered unlikely to occur for the low power SAWN^25^. The droplet size distribution plays an important role for applications, as for example in mass spectrometry large droplets can lead to loss of sensitivity and if not properly desolvated may lead to vacuum fluctuations, while small droplets will not.

**Figure 4:**
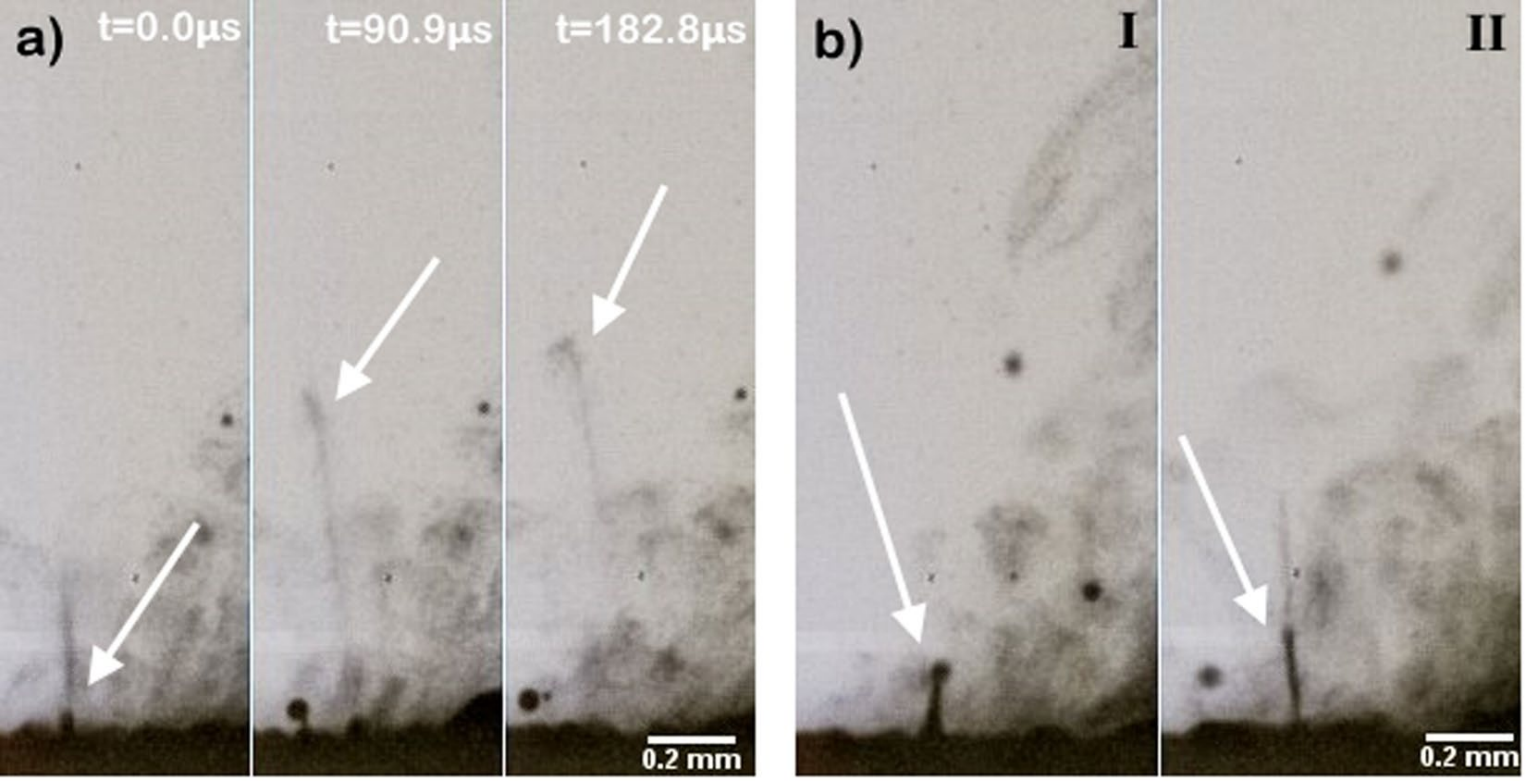
High-speed microscopic images from a nebulizing water droplet taken from the side of the SAWN chip. (**a**) Parametrically driven capillary waves are superposed on much larger waves set by the wavelength of the chip. The acceleration of these larger waves induces the sudden release of small droplets on their crests, visible here as jets, indicated by arrows. The three images are sequential, with time steps of 90.91 *μs* (11000 fps), showing that the jet containing small droplets is shot upwards. (**b**) The breakup of droplets is governed by the statics of the interference of waves, which sometimes can lead to the production of large amplitudes (ligaments) that subsequently break up to form droplets. Panel (b) shows two examples (I and II) of such waves in the case of the larger surface waves, i.e. not the parametrically excited waves which are too small to observe. Note that the two peaks shown in (**b**) are two extreme cases; most big droplets are created by waves of smaller amplitude.

We operate the SAWN chip with an arbitrary waveform function generator (RIGOL DG1062) amplified by a RF power amplifier (Mini-Circuits TVA-R5-13). As soon as the SAWN is operative, the droplet becomes opaque due to the waves on the surface of the droplet, but nebulization only starts after a certain waiting time. This waiting time decreases when the chip has been in operation longer, which is most likely due to heating of the chip and chip holder. This conjectured temperature-dependent onset of nebulization can be caused by lowering of the viscosity, which has a dampening effect; this is supported by the observation that a glycerol-water solution does not nebulize at the same applied power as pure water. After prolonged operation, heat (IR) camera images show that the chip and droplet can reach temperatures of around 70 °C. When the droplet starts to nebulize, a plume of micron-sized droplets is created together with much larger particles of ~50 *μ*m, visible in Fig. 3a. These larger particles are most likely due to the direct interaction of surface acoustic waves of the chip with the droplet, which have a much larger wavelength than the parametrically excited capillary waves. High-speed microscopic picture sequences of a nebulizing water droplet (Fig. 4a), suggest that the breakup of the capillary waves, which are believed to be superposed on the larger surface waves, is triggered by the rapid acceleration that these waves experience. The breakup of these droplets is therefore observed as jets (Fig. 4a) on top of the crests of these waves as illustrated by Fig. 1c. To the best of our knowledge, this is the first report of such a breakup mechanism for small droplets of the SAWN, or in fact for any type of nebulizer.

The breakup of droplets by Faraday waves is governed by the statistics of the interference of waves. An interesting large scale example of interference leading to very large amplitudes, are ocean freak waves or rogue waves. These waves that are much heigher than the average wave, pose a serious threat even to modern ships. Although the situation here is very different, extreme amplitudes do appear for which two examples are shown in Fig. 4b(I and II). Note that these are the larger surface waves, not the parametrically excited waves, which are too small to be observed.

### Nebulizer chip

The nebulizer chip (‘Grove’ type water atomizer by Seeed Technology) is a low-frequency (105 ± 5 kHz), low-power (2 W) and low-cost type of atomizer (Fig. 2). The transducer has a coin shape with and a rubber ring for isolation and a small reservoir to contain the fluid to be nebulized. If the transducer is placed in a small bath of water, water will be absorbed from under the chip into the reservoir such that the nebulizer can work continuously. In the middle of the chip there is a small bump where the nebulization takes place (Fig. 2b).

### Mist maker

The mist maker is a widely commercial available type of nebulizer mostly used for decoration and terraria (Fig. 2a). Unlike the other nebulizers investigated, this type is submerged. We immersed a Nebler nebulizer in water, placing it about 4 cm under the water surface where it produces a small cone-shaped fountain accompanied by a large amount of micron-sized droplets, seen as mist (Fig. 3c). The depth of the transducer follows from the fact that when the nebulizer is placed near the surface, it produces a lot of splashing, while if it is placed to deep, attenuation of the waves will reduce its effectiveness. The frequency of the nebulizer is 1700 ± 50 kHz with a power of 30 W.

## Experiments

All the droplet size distributions were measured by a laser diffraction method (Malvern Spraytec). Since the diffraction angle is inversely proportional to the droplet size, analysis of the scattered laser light intensity allows for the determination of the droplet size distribution, assuming a spherical droplet shape, an assumption easily met for such small droplets.

With dense sprays, multiple scattering events take place for which the software accounts for. There is however a limit in the density of the fog at which the droplet size distribution can still be measured accurately. Therefore, the location of the laser beam through the mist is chosen such that the mist is dilute enough (i.e. high transmission), but not too far from the nebulization location to prevent possible changes to the size distribution due to coalescence events or evaporation. It is know for example that when droplet sizes are micron sized, even at a relative humidity of 100%, distributions can change quickly over time, causing a significant change in the median droplet size^26^. Therefore the SAWN and nebulizer chip were placed in a glass container with a relative humidity of a 100%, with the walls of the container placed under an angle with the laser to prevent reflection of the beam on the detector. Background calibration and laser alignment are done between each measurement, thereby taking into account the walls of the glass container. The laser beam was placed 10 *cm* above the nebulizer, which appeared to be the optimum place of measurement, since it is high enough to have good transmission, yet not so far that the time between droplet creation and measurement is getting too long. For the mist maker measuring droplet sizes is slightly more complicated. The mist maker produces a large volume of droplets that do not ascent in the same manner as the other nebulizers. Directing the droplets through the beam with an airflow is likely to affect the droplet sizes by coagulation and evaporation. To introduce the least disturbance we placed the nebulizer in a beaker filled for roughly 3/4 with water, that after some operation time quickly reaches a relative humidity of a 100% above the water surface. The mist could then be guided directly through the laser beam by tilting the beaker, without the mist traveling a significant distance for any evaporation to take place. Even though the result does not show any abnormalities, this method is not optimal. No method however, can guarantee the absence of any coalescence or evaporation.

For the SAWN, we explored several waveforms such as pulsed, sinusoidal, square and AM-signals. There seems to be no affect of the waveform on the nebulization process. The only essential component seems to be the total power; certain waveforms give more power, resulting in better and quicker nebulization.

## Droplet Size Distributions

What determines the droplet size distribution in different types of fluid breakup, such as spray and jets, is well understood. In fluid fragmentation, where ligaments are of equal size, the rescaled droplet size distribution is given by a Gamma function ${\rm{\Gamma }}(x=d/\langle d\rangle ,n)$, where $\langle d\rangle$ is the mean droplet size and *n* a parameter that sets the ligament corrugation. For systems with highly corrugated ligaments, the distribution is broad and *n* = 4–5, while for very smooth ligaments $n=\infty$, which would result in a delta peak. For more complicated fluid breakup, such as in agricultural sprays, the ligaments themselves can also be distributed in size^21^, leading to a two-parameter compound Gamma distribution:

3 $${{\mathscr{P}}}_{m,n}(x=\frac{d}{\langle d\rangle })=\frac{2{(mn)}^{\tfrac{(m+n)}{2}}{x}^{\tfrac{(m+n)}{2}-1}}{{\rm{\Gamma }}(m){\rm{\Gamma }}(n)}{{\mathscr{K}}}_{m-n}(2\sqrt{mnx}),$$

where *m* sets the width of the ligament size distribution, *n* the ligament corrugation and ${{\mathscr{K}}}_{m-n}$ is a Bessel function of order *m* − *n*^27^. Here we argue that the ultrasonic atomization is similar to other fluid fragmentation processes, since here we can have a spread in ligament sizes due to a spread in wavelengths (Fig. 1b), and the pinch-off ligaments can be either smooth or rough.

**Figure 5:**
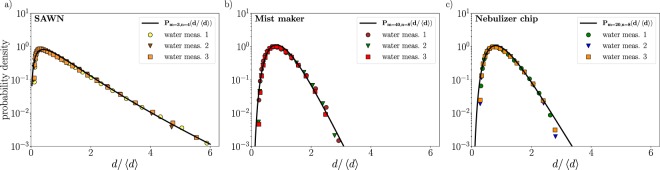
Rescaled droplet size (*d*) distributions for the three different nebulizers, each of which measured three consecutive times. Solid lines are fits to a two-parameter compound Gamma distribution ${{\mathscr{P}}}_{m,n}$, Eq. (3). (**a**) Size distribution of the SAWN for the smallest droplets, with fit parameters $m=3$ and $n=4$. The fit parameters indicate a very large spread in pinch-off ligament sizes and very corrugated ligaments, which can be expected for the nebulization of a droplet by the violent mechanism as seen in Fig. 4a and illustrated in Fig. 1c. Median droplet size for the smallest droplets of the SAWN is 1.1 *μ*m. (**b**) Size distribution for the mist maker with fit parameters $m=40$ and $n=8$ and a median droplet size of 5.6 *μ*m. These parameters indicate that the wavelengths are very similar and the pinch-off ligaments reasonably smooth, in accordance with the capillary wave mechanism of a free surface such as present in case of the the mist maker. (**c**) Size distribution of the nebulizer chip, with $m=20$ and $n=8$ and a median droplet size of 9.5 *μ*m. This shows that the nebulizer chip also has similar-sized pinch-off ligaments, although less so than the mist maker.

Figure 5 shows the rescaled size distributions for the different nebulizers for three consecutive measurements. The laser diffraction method does not allow for error bars on the data points, the setup averages hundreds of measurements per run, and the standard deviation between such measurements is typically small. The agreement between the three different runs shows however there is little variability between measurements. The droplet sizes are fitted with Eq. (3) to obtain the parameters *m* and *n*. Due to the form of Eq. (3), exponents can become large, leading to numerical problems in evaluating the distribution function. Standard minimization algorithms therefore do not work properly, since they are sensitive to the values of the initial guess. Fit parameters were therefore obtained by manually fitting the distribution function. This however does not affect the results, since minor changes of the parameters does not alter the conclusions in any way.

All distributions appear to be quite narrow, except for the smallest droplets of the SAWN. The values *m* and *n* for the SAWN distribution indicate large variations in wavelengths and rough ligaments. This is an expected result, considering that the smallest droplets of the SAWN are released by the rapid acceleration of the larger surface waves, thereby releasing small droplets in the form of jets as shown in Fig. 4a and schematically illustrated by Fig. 1c. Since this is a sudden and powerful ejection of droplets, there will be large variations in the ligament sizes with irregular pinch-offs.

The mist maker produces capillary waves on a free surface; its operation therefore bears the most resemblance to the classical Faraday wave scenario, where the breakup is smooth with similar-sized wavelengths (Fig. 1a). The distribution obtained experimentally is indeed the most narrow of all three nebulizers, with $m=40$ and $n=8$. Still, some randomness is expected; the mist maker creates a fountain of water at the center due to the driving force of the speaker, undoubtedly causing irregularities.

For the nebulizer chip, a layer of fluid is placed on the center of the transducer, where the nebulization takes place. The nebulizer only operates when the liquid layer is sufficiently thin, which can be associated with the shallow wave regime. We find that the size distribution is quite narrow with $m=20$ and $n=8$, which is somewhat surprising, considering that its operation appears to be similar to that of the SAWN. Still, what is distinctively different is that for the SAWN the smallest droplets are created by interaction with waves that are of a much larger length scale. Since the formation of the small and big droplets of the SAWN are interconnected, the lack of such bigger droplets implies that the nebulization mechanism for the nebulizer chip must be different.

**Figure 6:**
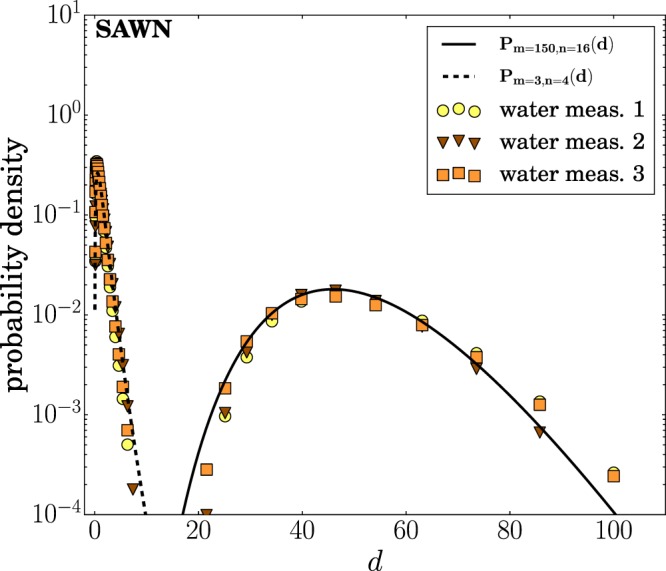
Droplet size distribution of the SAWN including the larger droplets. These larger droplets are due to waves created by the direct interaction of the surface acoustic waves and the droplet. Three measurements are shown along with the fit (solid line) according to Eq. (3).

The SAWN creates not only micron-sized droplets but also much larger (~50 *μ*m) droplets that are due to the direct interaction of the surface acoustic waves with the droplet on the chip. During nebulization, the edge of the droplet flattens out, creating a thin fluid layer. There, as a result of constructive interference, occasional peaks arise that frequently break up to form large droplets and simultaneously create the previously described jets of small droplets (Fig. 4). In contrast to what is seen for the smallest droplets, the big droplets are narrowly distributed (Fig. 6). With $m=150$ and $n=15$, the waves responsible for droplet formation seem to be of equal size with reasonably smooth pinch-off ligaments. The sizes of the peaks are determined by the precise statics of the interference of such waves, which apparently are quite regular. Although Fig. 4b) shows two examples of peaks, it should be noted that these are two extreme cases of which no conclusion can be drawn about the general shape of the pinch-off. Most spikes that lead to the formation of big droplets are much smaller and barely visible due to masking by other waves and smaller droplets.

## Droplet Sizes

In Table 1 the median droplet sizes for the different types of nebulizers are listed, together with the ratio *D*_50_/*λ*, where *λ* is the capillary wavelength. This ratio is the proportionality constant $\kappa$ in Eq. (2), which Lang^2^ found to be 0.34 in the case of a *number* median. Although the investigated devices are distinctively different, with frequencies almost 2 orders apart, we find proportionality constants in the range 0.17–0.65, close to Lang’s value. The proportionality constants of the nebulizer chip and the big droplets of the SAWN are both at the lower end of this range. This could be because the assumption of a sufficient height of the fluid layer is not met for these cases: for shallow water, one expects smaller waves, resulting in a smaller proportionality constant.

**Table 1:** Frequency, median diameter and ratio of median diameter over capillary wavelength, calculated with Kelvin’s formula.

Device	Frequency	*D*_50_ (*μm*)	*D*_50_/*λ*
SAWN small droplets	9.6 MHz	1.1	0.39
Nebulizer chip	105 ± 5 kHz	9.5	0.17
Mist maker	1700 ± 50 kHz	5.6	0.65
**Device**	**Frequency**	***D***_**50**_ **(** ***μm*** **)**	***D***_**50**_ **/** $\ell$
SAWN big droplets	—	56	0.16

For the big droplets produced by the SAWN, the wavelength $\ell$ of the surface acoustic waves of the chip is used, which is set by the distance of the metallic strips of the IDT.

## Conclusions

This is the first study that focuses primarily on the droplet size distribution of ultrasonic nebulizers. We provide a new view on the droplet sizes, by employing a distribution function that can be directly linked to the breakup mechanisms. This is in contrast with other studies, where distribution functions are fitted without any clear physical interpretation. Our proposed interpretation of the fit parameters of the distributions could give insight in the waves that contribute to droplet formation, something that is otherwise difficult to assess by direct measurements, due to the small length- and time-scales involved. Additional measurements on systems with larger Faraday waves, could further develop this notion and allow for a direct verification.

We investigated three types of ultrasonic nebulizers and show that the droplet size distribution is analogous to that observed for other types of fluid breakup such as sprays. We argue that the shape of the distribution is set by the dispersion in wavelengths and the roughness of the ligaments. In the classical case of ultrasonic nebulization through the capillary wave mechanism, wavelengths are equal in size and the pinch-offs are smooth, leading to a narrow size distribution. Our results therefore support this classical picture of nebulization for some types of nebulizers. However, our results also reveal that droplets can be very different in size, something that has not has not been observed before. For a nebulizer based on surface acoustic waves, there are waves of two wavelengths involved: waves on the order of the surface acoustic waves and the much smaller parametrically excited capillary waves. We theorize that as a result of the fast acceleration of these larger waves, the smaller waves break apart, thereby ejecting droplets from the surface. This phenomenon is observed as jets of small droplets on top of the crests of these waves. Since according to this view droplets are shed from the surface in an abrupt way, large variations in ligament size can be expected, leading to a big dispersion in droplet sizes.

For different devices that operate at different frequencies, the capillary wavelength scales well with the the median droplet size, with proportionality constants of order unity. In some cases the liquid layer is shallow, which could explain the smaller droplets we observe for these nebulizers. In this work we did not investigate the effect of the droplet size on fluid properties such as viscosity, surface tension and density, which would be necessary to systematically evaluate the relation between droplet sizes and capillary wavelength. Even though most existing scaling laws predict the fluid parameters to have only a minor effect on the median drop size, their effect on the droplet size distribution would also be of interest.
